# Snail1 controls cooperative cell plasticity during metastasis

**DOI:** 10.18632/oncoscience.262

**Published:** 2015-11-16

**Authors:** Josep Baulida

**Affiliations:** Programa de Recerca en Càncer, Institut Hospital del Mar d'Investigacions Mèdiques (IMIM). Parc de Recerca Biomèdica de Barcelona (PRBB). C/Dr. Aiguader, Barcelona

**Keywords:** Snail, metastasis, EMT, CAF, tumor-stroma

Mortality in cancer is strongly associated with the capacity of tumor cells to spread and critically affect other tissues and organs. Genetic mutations accumulated by tumor cells and cross-signaling between tumor and host cells underlie the formation of metastasis. Cancer-activated fibroblasts (CAFs), which are host fibroblasts activated by tumor signaling, can alter tumor cell behavior by both paracrine signaling (secreting diffusible molecules) and mechanical signaling (modifying the composition and organization of the stroma). These fibroblasts resemble myofibroblasts (MFs) of the granulation tissue generated during wound healing, which produce a rigid desmoplastic stroma rich in signaling molecules and cross-linked extracellular fibers. Desmoplasia favors malignant tumor cell properties such as mobility, stemness, and even resistance to pharmacological insults [1].

Our research group has been studying the role of Snail1 on tumor progression, a transcription factor involved in the epithelial-to-mesenchymal transition (EMT). EMT is a plasticity process by which epithelial cells exchange their structural determinants for mesenchymal ones. Thus, EMT promotes a transition from a static phenotype with apico-basal polarity towards a motile one with anterior-posterior polarity. EMT provides a simple explanation of how tumor cells escape from the primary tumor, via Snail1. Classically, Snail1 has been described as a transcriptional repressor of epithelial genes, particularly of those affecting the cell architecture. Thus, Snail1 expression in epithelial cells represses proteins in epithelial junctions, such as E-cadherin and claudin/occludin, and in epithelial intermediate filaments, such as cytokeratin 18, which then initiates EMT [2]. In tumors, EMT is incomplete because the majority of tumor cells that have gained mesenchymal markers have also retained some epithelial determinants. In this not-fully-differentiated state, cells acquire stem properties that allow them to behave as tumor-initiating and drug-resistant cells. In fact, the cancer stem cell (CSC) phenotype is comparable to a partial EMT status and is strongly dependent on signaling from the tumor stroma.

Recent data indicate that Snail1 is also required for the trans-differentiation of fibroblasts. Indeed, in mammary and colonic tumors, Snail1-positive CAFs are more easily detected by histological studies than Snail1-positive tumor cells [3]. In these fibroblasts, the actions of Snail1 cannot be attributed to the repression of epithelial determinants, as these are constitutively repressed. Rather, Snail1 is required for the transcription [4] and the polymerization of extracellular molecules [3] involved in desmoplasia. Fiber polymerization is mediated by of RhoA, a GTPase activated by tumor secreted factors such as TGFβ in a Snail1-dependent manner. Thus, Snail1 is required for the assembly of αSMA-reinforced acto-myosin fibers and for the tensional activity at focal adhesions to polymerize extracellular fibronectin [3]. So far, the molecular mechanism linking Snail1 and RhoA has not been addressed.

In addition to affecting epithelial and mesenchymal cell architecture, Snail1 has recently also been reported to control the paracrine potential of both CAFs and tumor cells. Specifically, the cytokine profile secreted by CAF lines established from colon cancer patients, and their capacity to induced migration of colon tumor cell in a paracrine manner, was found to depend on the levels of Snail1 expressed by the CAFs [5]. In tumor epithelial cells, TNFα induces the transcription of secreted factors, such as CCL2 and CCL5, through the action of acetylated Snail1 [6].

Although molecular events promoted by Snail1 differ for each cell type, current experimental data indicate that Snail 1) controls the cell architecture of both epithelial tumor cells and mesenchymal host cells and 2) regulates the paracrine and mechanical signaling between tumor and host cells, thereby modulating metastasis formation. Thus, in tumor cells, expression of Snail1 cells can promote partial EMT and the essential metastatic properties of stemness and motility. However, in epithelial tissues, adherens junctions generate a repressive Snail1 feed-back loop that restricts its expression [2]; therefore, only those specific cells that are within an adequate mechanical niche and receive the appropriate paracrine stimuli can overcome the threshold restrictions and undergo partial EMT. In contrast, in CAFs, a positive feedback loop to Snail1 and RhoA activity assures a myofibroblastic phenotype. This loop include focal contacts of CAFs that can mechano-sense the desmoplastic extracellular matrix that they generate [7]. In this way, fibroblasts initially activated by paracrine signaling from tumor cells can fix their phenotype and provide Snail1-dependent biochemical and biomechanical signaling to promote metastasis. It is now clear that Snail1 contributes to tumor progression in a much more potent manner than initially proposed, by regulating both the plasticity of tumor and tumor-activated cells as well as the cross-signaling between them.
